# Lipid transfer protein: a link between food and respiratory allergy

**DOI:** 10.1186/2045-7022-3-S3-P58

**Published:** 2013-07-25

**Authors:** J Sanchez-Lopez, L Tordesillas, A Diaz-Perales, M Pascal, M Rueda, R Muñoz-Cano, R Vilella, A Valero, C Picado, J Bartra

**Affiliations:** 1 Pulmonology and Respiratory Allergy. ICT., Hospital Clinic, Barcelona, Spain; 2 Departament of Biothecnology, ETS Ingenieros Agronomos. Universidad Politécnica de Madrid, Madrid, Spain; 3 Immunology, Hospital Clinic, Barcelona, Spain

## Background

Lipid transfer proteins (LTP) are considered true food allergens because sensitization takes place in the gastrointestinal tract, in contrast with other food allergens like profilins or Bet v 1-homologues, where sensitization is a consequence of an allergic sensitization to inhaled allergens. Although allergy to peach LTP (pru p 3) is frequently associated to sensitization to plane tree pollen LTP (Pla a 3) and/or mugwort LTP (Art v 3), it has never been elucidated if this cross-reactivity could lead to a respiratory allergy. The aim of this study is to demonstrate that a LTP-dependent food allergy with the adequate exposure can induce a LTP-dependent respiratory allergy.

## Methods

Two groups of patients were selected: group A, patients allergic to Pru p 3 and sensitized to Art v 3, and group B, controls not sensitized to Pru p 3 nor Art v 3.

Skin prick test (SPT) and specific IgE (sIgE) to peach, mugwort, Pru p 3 and Art v 3, and nasal challenge test (NCT) to Art v 3 and mugwort were performed. NCT was controlled by acoustic rhinometry, visual analogue scale (VAS) and total nasal symptom score (TNSS).

*In vitro* tests included leukotrienes (cysLT) detection in nasal lavage before and after NCT, immunoblotting with mugwort pollen extract, ELISA and ELISA inhibition with Pru p 3 and Art v 3.

## Results

13 patients and 9 controls were selected. The median of nasal volumes at group A decreased significantly from 100% (baseline value) to 77.5% 15 minutes after NCT with Art v 3 (*P*<0.001), TNSS and VAS significantly changed (*P*<0.001) from baseline to 15 minutes after NCT. CysLT levels were significantly increased in nasal lavage 15 minutes after NCT from 1784.38 pg/ml to 4493.21 pg/ml (*P*<0.001). Immunoblotting with complete extract of mugwort showed that 8 patients from group A had only a band around 10 kDa, presumably corresponding to Art v 3. These patients were challenged with a complete mugwort pollen extract, with a positive response in all of them.

The ELISA experiment showed higher detection of Pru p 3 than Art v 3 in all cases, and ELISA inhibition with Pru p 3 and Art v 3 indicated that Pru p 3 was a stronger inhibitor than Art v 3, highlighting that the primary sensitizer was Pru p 3.

## Conclusion

Patients allergic to Pru p 3 who present a sensitization to Art v 3 by a cross-reactivity phenomenon, can elicit symptoms when exposed to mugwort pollen. That means that a food allergy induced by digestive route may lead to a respiratory allergy when exposed to a LTP-containing pollen.

## Disclosure of interest

None declared.

